# Supplementary material for: Attitudes of medical students on conflict of interest: A comparative study of Korea and France
Source: PLoS One. 2023 Nov 2;18(11):e0293808. doi: 10.1371/journal.pone.0293808 (PMC10621834; doi:10.1371/journal.pone.0293808)
Supplement: Attachment — Submitted filename: Korean medical student attitudes on COI.docx [file pone.0293808.s002.docx]

Korean medical student attitudes on COI

PlosOne

Synopsis

This paper replicates a French study looking at attitudes and perceptions amongst medical students around conflicts of interest. It provides results of a survey of students and junior doctors about their reported understanding and views about conflict of interest and their experiences interacting with pharmaceutical industry representatives.

Overall comment

This is a useful paper and provides important information to document knowledge and attitudes about COI amongst Korean medical students/junior doctors. As far as I am aware this is the first paper to report on this topic in Korea. I commend the authors for reporting on this and look forward to seeing it add to the literature. We need these kinds of baseline papers in order to assess interventions to reduce the influence from industry on healthcare. I have some minor comments about wording and presentation, I hope the authors can address these without too much difficulty, to make this a more readable paper. I also suggest the authors are more circumspect in their claims about the degree to which their survey response is representative of the target population since they do not know the numbers in their target audience.

Specific comments:

INTRODUCTION

P3, line 39 / para 1	I do not understand the concept of ‘rebates in pharmaceutical sales’. Can the authors elaborate on the current system in Korea to which this refers? What are the rebates / who gets them / who gives them / how are they awarded etc.

P4, line 50 onwards / para 1	The authors’ representation of Etain et al’s paper was confusing. I think the syntax needs review. For example, it reads as if Etain et al were exposed to pharma reps a lot and had contradictory ideas about bias. I think the authors mean to say that Etain et al found that medical students were exposed etc.

P4, line 55 / para 3 The authors call this a qualitative survey (also on p 16) but it is a quantitative survey. They also say they compare attitudes amongst students, but they also compare knowledge, education, experiences with pharma reps etc.

P4, lines 59-63 / para 4	I am not sure this paper needs an ‘hypothesis’ approach. This paragraph could be reworded to simply present the aim.

Overall the introduction could benefit from some more intensive introductory discussion about COI amongst medical students / doctors – eg why this topic is important and worth studying. do eg explain why we care about it, what are the potential harms, what is the evidence of influence, what do we even mean by the term COI. There is much useful evidence on this topic that the authors could make use of – for example the authors might like to explore the reference list in a previous publication of mine on this topic

PARKER L (2019) I’m more susceptible to drug company money than I’d like to be. *British Medical Journal*, 19 December. Available at: <https://blogs.bmj.com/bmj/2019/12/12/lisa-parker-im-more-susceptible-to-drug-company-money-that-id-like-to-be/>

METHODS

The authors recruit students and junior doctors, but throughout the paper typically use the term ‘students’. I suggest adding a line to explain that the term student will be used to mean all the participants unless otherwise specified, so the readers are clear.

P5, line 80-81 / para 4	The authors do not make any kind of claim that they wrote to all medical schools or all resident institutions. They do not know the exact number who received the survey so cannot supply a response rate. Given all this, I suggest there is no substance to the claim they make in the Discussion that this represents the views of all Korean students. Nevertheless, their survey response numbers are substantial and this remains a very worthy study. I suggest the authors try to give some kind of context to their numbers to help readers who are unfamiliar with Korea – eg, how many medical schools are there in Korea, and can they estimate approximately how many residents there are (eg maybe the Medical Registration Board would assist?) or if those figures are unavailable, maybe estimate how many resident institutions there are?

RESULTS

P6, line 94 / para 3	The authors write ‘although [students] expressed interest in whether the courses they attended concerned COI …’ I am not sure what this means – can the authors expand?

P6, lines 96-100 / para 4	This paragraph lists ratios of items, I don’t understand what this means, what is the ratio the authors are referring to? Do they just mean % of students who answered ‘yes’ to each question?

P8, line 114 / para 1	The authors report that ‘35.5% in Set 1 and 45.6% in Set 2 thought monetary offerings generated a COI’. A similar statement is repeated on p9,line 120 / para 1 and on p 13, line 152/para 1. I don’t understand this statement. I thought Set 1 and Set 2 were just groupings of questions, not groupings of participants?

P11, line 142 / para 2	I suggest EDITING to read: “determine whether personal research on COI or lecture participation in COI affected BELIEFS ABOUT IMPACT OF COI ON future prescriptions BY SELF OR OTHERS more.”

Table 3	I found this table very hard to interpret, and the summary sentence after it (beginning “the analysis found that the P-value…”) was also hard to understand. Can the authors please clarify the meaning of this table, perhaps providing the results in a different format would help?

Table 4	I suggest the authors specify “Korean medical students” in the title, and can then delete the row of K K K K. This could be repeated in Table 5. I don’t understand the point of providing a P-value in this table or Table 5. I can’t see that it is relevant whether or not there is statistical significance between the ‘yes/no/don’t know’ groups.

P 13, line 158	“The degree to which it affects…” Can the authors explain what they are talking about here?

P14, line 167 / para 3	I suggest the following EDITS: “Regarding the STUDENTS’ PERCEPTION ABOUT possibility of bias…”

DISCUSSION

P 15, line 179 / para 4	I suggest omitting this presentation of the paper as an hypothesis approach, as mentioned earlier.

P 15, line 182 / parka 5	Can the authors clarify their meaning: “We met the minimum study population of 95% confidence level …”

P 16, line 185-188	As mentioned above, I don’t think the authors can claim that this survey represents the opinions of Korean medical students

P 16, line 200 / para 5	The authors suggest students should spend more time on quality education about COI, but I wonder whether this is ignoring the responsibility of the educational institutions to encourage accurate and interesting education for students. Can the authors provide any evidence / references about this.

P 17, line 17 / para 1	I really would like to see some stronger referencing of the literature on COI and suggests about systemic interventions that might reduce the influence of pharma reps / industry on students. This should go beyond individual disclosure / mandated transparency and might include topics such as : hospital / university policies to reduce pharma rep access to students, hospital / industry policies to limit the type of pharma rep gift giving and the amounts involved, university and hospital policies to limit the use of teachers who accept pharma industry gifts etc etc. Again, I encourage the authors to read widely around the topic of COI and how to reduce it so that they can provide a stronger argument and more useful suggestions for how Korea might address this issue in its healthcare system.
