# Supplementary material for: Attitudes of medical students on conflict of interest: A comparative study of Korea and France
Source: PLoS One. 2023 Nov 2;18(11):e0293808. doi: 10.1371/journal.pone.0293808 (PMC10621834; doi:10.1371/journal.pone.0293808)
Supplement: Attachment — Submitted filename: 1. Response to Reviewers (Revision).docx [file pone.0293808.s003.docx]

**Response to Reviewers**

**Reviewers' comments:

Reviewer #1:**

Thank you very much for your study
I have some comments that I list in chronological order

**Thank you so much**

-------------------------------------------------------------------------------------------------------------

1. Line 27: “lecture participation in COI affected future prescriptions more” is unclear

**Given the modifications made to the contents and format of Table 3, the corresponding comment section has been updated as follows.:**

**“****As students moved up to higher education courses, they had greater interaction with pharmaceutical industry representatives, received more gifts, and displayed an increased likelihood of acknowledging the influence of COI on biases.” [Revised Manuscript Line 29-32]**

-------------------------------------------------------------------------------------------------------------

2. Line 50: “he was exposed to pharmaceutical sales representatives a lot, and he had a contradictory idea that he would not be biased, although others could be” is unclear: one could understand that Etain himself is exposed to pharmaceutical sales representatives while its conclusion concerns students.

**We revised the sentence as follows:**

**“In addition, many students were exposed to pharmaceutical representatives, and they held the contradictory belief that biases could affect others but not themselves.” [Revised Manuscript Line 86-88]**

-------------------------------------------------------------------------------------------------------------

3. Line 70: “a questionnaire was distributed to 10 students in advance to evaluate their 70 understanding and reflect their opinion on the subject”. Did you modify the questionnaire according to this first evaluation?

**First of all, “70” is a number that means line number, and the subjects of the first evaluation were 10 students.**

**In addition, the following Korean expressions were clearly incorporate some of the opinions from the initial evaluation. For instance, there were suggestions to explicitly mention the gift or financial support provider (pharmaceutical laboratory) and to convey the intended meaning of the phrase ('impression of “being bought"') more clearly.**

-------------------------------------------------------------------------------------------------------------

4. Line 79: “The potential participants » : can you precise how many potentials participants were expected?

**"The potential partners" means the total sample size of this study.**

**The number of subjects in this study is shown in the following table.**

| **classification** | | **Num. of person** |
| --- | --- | --- |
| **Medical**  **residents*** | **Intern** | **3,773** |
|  | **Resident 1** | **3,149** |
|  | **Resident 2** | **3,299** |
|  | **Resident 3** | **3,273** |
|  | **Resident 4** | **2,108** |
| **Medical**  **students**** | **pre-medical** | **10,819** |
|  | **medical** | **5,719** |
| **Total** | | **32,140** |

*** Ministry of Health and Welfare / Committee for Assessment of Training Conditions**

**: Quota of Intern & Resident in 2021**

**** Ministry of Education / Center of University Information Disclosure**

**: Current Medical Students in July 21, 2021**

**The number of subjects in the above is not arbitrarily determined by the author but is based on data released by relevant public institutions.**

-------------------------------------------------------------------------------------------------------------

5. Line 93: “Although they expressed interest in whether the courses they attended concerned COI (54.6%), about half said the professors teaching the courses did not disclose their COI information (46.4%)”: I think that these ideas are not related and should not be in the same sentence.

**The questionnaire corresponding to the question is as follows.**

**4.6. I would like to know the COI of my teachers when they teach me?**

**1) Yes (54.6%)	2) No	3) Don't know**

**4.5. Do your teachers mention their COI during their lessons?**

**1) Don't know	2) No (46.4%)	3) Some	4) All**

**We agree with you.**

**While the question holds individual value, I realized that it is inappropriate to express it in the same sentence since 'professor' and 'course' are not specified.**

**We revised the sentence as follows:**

**“A large number of students reported insufficient education on COI (63.7%) and reported infrequent attendance in lectures (8.5%) or personal research (10.3%). Additionally, over half of the respondents (54.6%) expressed interest in COI information regarding their courses.” [Revised Manuscript Line 131-134]**

-------------------------------------------------------------------------------------------------------------

6. Lines 105 to 107: the 51 students are probably the same in the 2 part of the paragraph but can you precise?

**The questionnaire corresponding to the question is as follows.**

**3.2. During your studies, do you feel that you received enough information about declaration of COI?**

**1) Yes	2) No	3) Don't know**

**3.3. During your studies, did you receive a lecture or a tutorial on COI?**

**1) Yes	2) No**

**2.1. Do you think you know how to define what a COI is?**

**1) Yes	2) No	3) I'm not sure**

**There were 35 respondents who answered 'Yes' to question 3.2, 33 respondents who answered 'Yes' to question 3.3, and 17 respondents who answered 'Yes' to both questions.**

**Hence, there were 51 unique respondents who answered 'Yes' after excluding duplicate responses (35+33-17=51). Additionally, out of these 51 respondents, 23 answered 'No' or 'Don't Know Actually' to question 2.1.**

**Therefore, We believe the analysis result is accurate.**

-------------------------------------------------------------------------------------------------------------

7. Table 1: can you precise that Etain and al wanted to investigate the Medical Students’ Knowledge of Situations at Risk of COI according to their level within medical school (and so did you)?

**The previous research paper by Etain et al. describes the analysis results based on the curriculum level. The author of this paper also intended to conduct a comparative study using the findings from previous studies, so We think so.**

-------------------------------------------------------------------------------------------------------------

8. Line 120 and 121: can you precise what this percentages (ie: 34.5 and 71.7) refer to?

**The average score for Set 1 among French students is 34.5% [(27.7+32.9+35.0+35.6+41.5)/5=34.5%]. This value was found to be similar to the average score for Set 1 among Korean students [(30.9+39.7+45.1+26.5+35.1)/5=35.5%]**

**The average score for Set 2 among French students is 71.7% [(56.0+58.7+69.2+85.5+89.1)/5=71.7%]. It was observed that there is a significant difference from the average score for Set 2 among Korean students [(43.8+49.2+25.8+41.8+67.3)/5=45.6%]**

-------------------------------------------------------------------------------------------------------------

9. Line 124: “but Korean students showed a large difference” the study is not designed to show a difference. Moreover p values are statistically significative in both study: I think that this important point deserve clarification

**We agree with you**

**Therefore, We intend to address these points in the Discussion section.**

**“****While each study conducted in France and Korea was not specifically designed to demonstrate differences, it would be meaningful to compare the research findings between the two countries using a consistent approach to validate any potential disparities.” [Revised Manuscript Line 232-234]**

-------------------------------------------------------------------------------------------------------------

10. In table 2, in spite of the impossibility of statistical comparison, there is a marked difference between France and Korea in exposure to marketing strategies: do you have any explanation (within the discussion part)?

**As with the response to the previous comment (9. Line 124), We believe it is meaningful to compare the results of each study conducted in France and Korea using a similar approach to identify any differences.**

**Therefore, We intend to address these comments in the Discussion section.**

-------------------------------------------------------------------------------------------------------------

11. The data within tables 4 and 5 are original and were not in the study of Etain and al: perhaps it deserves more comments in the discussion?

**We believe that the knowledge about COI is related to the importance of education. Therefore, even though it was not included in the previous study, we have included it as an additional aspect.**

**However, not only does the format match that of Tables 1 and 2, but there is no issue with conveying the meaning through a description instead of using tables. Therefore, Tables 4 and 5 will be omitted, and their contents will be discussed in the following section.**

**“Further analysis revealed that respondents who believed they were knowledgeable about COI (50.1%) showed a greater awareness of COI compared to those who believed they lacked knowledge (31.8%). Additionally, they exhibited a higher awareness of direct COI (56.0%) compared to indirect COI (44.1%), as shown in Table 2.” [Revised Manuscript Line 216-219]**

-------------------------------------------------------------------------------------------------------------

12. Line 165: this sentence should be moved from the results section to the discussion and probably deserve a larger analysis regarding this paradoxical result.

**We agree with you.**

**We will move this sentence to the Discussion section and mention that further analysis is needed as a follow-up study.**

**“Further analysis revealed that respondents who believed they had knowledge about COI (18.9%) reported a higher incidence of receiving gifts compared to those who believed they lacked knowledge (3.0%). On the other hand, these results suggest that students with more exposure to pharmaceutical salespeople or receiving gifts had a better understanding of COI. Therefore, further analysis through follow-up studies is necessary.” [Revised Manuscript Line 254-258]**

-------------------------------------------------------------------------------------------------------------

13. Line 190: this sentence is unclear

**We revised the sentence as follows:**

**“and the residents evenly participate in 18 out of the 26 training departments operating in Korea. With the exception of the 5 training departments with a total quota of less than 50, almost all departments are involved.” [Revised Manuscript Line 246-248]**

-------------------------------------------------------------------------------------------------------------

**Reviewer #2:**
PlosOne

Synopsis
This paper replicates a French study looking at attitudes and perceptions amongst medical students around conflicts of interest. It provides results of a survey of students and junior doctors about their reported understanding and views about conflict of interest and their experiences interacting with pharmaceutical industry representatives.

Overall comment
This is a useful paper and provides important information to document knowledge and attitudes about COI amongst Korean medical students/junior doctors. As far as I am aware this is the first paper to report on this topic in Korea. I commend the authors for reporting on this and look forward to seeing it add to the literature. We need these kinds of baseline papers in order to assess interventions to reduce the influence from industry on healthcare. I have some minor comments about wording and presentation, I hope the authors can address these without too much difficulty, to make this a more readable paper. I also suggest the authors are more circumspect in their claims about the degree to which their survey response is representative of the target population since they do not know the numbers in their target audience.

-------------------------------------------------------------------------------------------------------------

I did not receive any supplementary files to review. It would be useful for the reader to have access to the survey questions.

**Thanks for your comment.**

**When We submit the 'Response to Reviewers' file, We will send you the 'Questionnaire' file together.**

-------------------------------------------------------------------------------------------------------------

Specific comments:

INTRODUCTION

(P3, line 39 / para 1) I do not understand the concept of ‘rebates in pharmaceutical sales’. Can the authors elaborate on the current system in Korea to which this refers? What are the rebates / who gets them / who gives them / how are they awarded etc.

**We revised it by adding the description as below :**

**“In general, rebates refer to the act or the amount by which a seller reduces the selling price by refunding a portion of the sale to the buyer to promote sales.**

**However, under the Korean Pharmaceutical Affairs Act, the pharmaceutical sales rebate refers to a 'kickback' wherein a doctor receives private profits in correspondence to the prescription and purchase of necessary drugs from a pharmaceutical company or supplier while prescribing drugs for medical treatment.**

**Types of pharmaceutical sales rebates announced by Korea's Health Insurance Review and Assessment Service (HIRA) include ① drug adoption case fees, ② drug prescription case fees, ③ regular advisory fees, ④ tourism or meal expenses, and ⑤ expenses related to overseas conference attendance.**

**According to the Ministry of Health and Welfare's 'Notification Status of Illegal Rebate Detection between 2015 and 2018,' it appears that illegal rebates have been gradually decreasing. However, it has been discovered that rebates are not actually decreasing, but rather that pharmaceutical companies are providing rebates through sales agencies known as CSOs (Contract Sales Organizations), resulting in their exclusion from the statistics. They are taking advantage of the fact that CSOs are not classified as drug suppliers under the Pharmaceutical Affairs Act, making it impossible to impose penalties even if they are caught.(1)” [Revised Manuscript Line 57-73]**

-------------------------------------------------------------------------------------------------------------

(P4, line 50 onwards / para 1) The authors’ representation of Etain et al’s paper was confusing. I think the syntax needs review. For example, it reads as if Etain et al were exposed to pharma reps a lot and had contradictory ideas about bias. I think the authors mean to say that Etain et al found that medical students were exposed etc.

**We revised the sentence as follows:**

**“In addition, many students were exposed to pharmaceutical representatives, and they held the contradictory belief that biases could affect others, but not themselves.” [Revised Manuscript Line 86-88]**

-------------------------------------------------------------------------------------------------------------

(P4, line 55 / para 3) The authors call this a qualitative survey (also on p 16) but it is a quantitative survey. They also say they compare attitudes amongst students, but they also compare knowledge, education, experiences with pharma reps etc.

**We revised the sentence as follows:**

**“In this context, we also conducted a survey of medical students in Korea to collect quantitative information on knowledge, education, personal exposure, and opinions about COI and to identify priorities for future education on COI.” [Revised Manuscript Line 92]**

**“and (2) it provides quantitative data on potential differences in perception according to curriculum stage, including among preclinical and clinical students.” [Revised Manuscript Line 252]**

-------------------------------------------------------------------------------------------------------------

(P4, lines 59-63 / para 4) I am not sure this paper needs an ‘hypothesis’ approach. This paragraph could be reworded to simply present the aim.

**We decided to understand and accept your opinion, and revised the sentence as follows:**

**“This paper aims to identify the following. : “ [Revised Manuscript Line 95-96]**

-------------------------------------------------------------------------------------------------------------

Overall the introduction could benefit from some more intensive introductory discussion about COI amongst medical students / doctors – eg why this topic is important and worth studying. do eg explain why we care about it, what are the potential harms, what is the evidence of influence, what do we even mean by the term COI.

There is much useful evidence on this topic that the authors could make use of – for example the authors might like to explore the reference list in a previous publication of mine on this topic

PARKER L (2019) I’m more susceptible to drug company money than I’d like to be. British Medical Journal, 19 December. Available at: <https://blogs.bmj.com/bmj/2019/12/12/lisa-parker-im-more-susceptible-to-drug-company-money-that-id-like-to-be/>

**We added a description of COI in the introduction section as below.**

**“There are many studies (2) (3)** **demonstrating the impact of pharmaceutical companies' marketing activities on doctors' prescribing practices. Consequently, pharmaceutical companies are compelled to engage in a range of marketing activities, both direct and indirect, in order to influence doctors' prescriptions.**

**Students are appealing marketing targets for pharmaceutical companies because established prescribing habits are not easily altered. As a result, medical students and junior doctors are susceptible to being exposed to conflicts of interest with pharmaceutical companies. (4)**

**A conflict of interest is a set of circumstances that creates a risk that professional judgment or actions regarding a primary interest(ex. the welfare of patients) will be unduly influenced by a secondary interest(ex. financial gain). (5)**

**Conflict of interest is a specific 'circumstance.' Therefore, while it may not be a problem in itself, it has a high likelihood of leading to biases. Hence, proactive measures are necessary to prevent it, and "disclosure" is widely recognized as a common solution. (6)” [Revised Manuscript Line 39-52]**

-------------------------------------------------------------------------------------------------------------

METHODS

The authors recruit students and junior doctors, but throughout the paper typically use the term ‘students’. I suggest adding a line to explain that the term student will be used to mean all the participants unless otherwise specified, so the readers are clear.

**We revised the sentence as follows**

**: In total, 388 medical students, interns and residents (hereafter referred to as ”students”). [Revised Manuscript Line 23]**

**Furthermore, the term 'medical students, interns, and residents' was consolidated into 'students' for consistency.**

-------------------------------------------------------------------------------------------------------------

(P5, line 80-81 / para 4) The authors do not make any kind of claim that they wrote to all medical schools or all resident institutions. They do not know the exact number who received the survey so cannot supply a response rate. Given all this, I suggest there is no substance to the claim they make in the Discussion that this represents the views of all Korean students. Nevertheless, their survey response numbers are substantial and this remains a very worthy study. I suggest the authors try to give some kind of context to their numbers to help readers who are unfamiliar with Korea– eg, how many medical schools are there in Korea, and can they estimate approximately how many residents there are (eg maybe the Medical Registration Board would assist?) or if those figures are unavailable, maybe estimate how many resident institutions there are?

**Although the paper did not mention the minimum number of subjects, the investigation included the number of medical students and residents to calculate the minimum number of subjects during the establishment of the research plan. The main contents are as follows.**

| **classification** | | **Num. of persons** | **Num. of Institutes** |  |
| --- | --- | --- | --- | --- |
| **Medical**  **residents*** | **Intern** | **3,773** | *** Institute of**  **medical**  **trainging**  **: 214** | *** Minimum number of**  **subjects : 380**  **- confidence level : 95%**  **- error range : 5%** |
|  | **Resident 1** | **3,149** |  |  |
|  | **Resident 2** | **3,299** |  |  |
|  | **Resident 3** | **3,273** |  |  |
|  | **Resident 4** | **2,108** |  |  |
| **Medical**  **students**** | **pre-medical** | **10,819** | *** College of**  **medicine**  **: 35** |  |
|  | **medical** | **5,719** |  |  |
| **Total** | | **32,140** |  |  |

*** Ministry of Health and Welfare / Committee for Assessment of Training Conditions**

**: Quota of Intern & Resident in 2021**

**** Ministry of Education / Center of University Information Disclosure**

**: Current Medical Students in July 21, 2021**

-------------------------------------------------------------------------------------------------------------

RESULTS

(P6, line 94 / para 3) The authors write ‘although [students] expressed interest in whether the courses they attended concerned COI …’ I am not sure what this means – can the authors expand?

**The author did not intend to arbitrarily expand the meaning, but rather based it on the analysis results of the survey response status.**

**The questionnaire corresponding to the question is as follows.**

**4.6. I would like to know the COI of my teachers when they teach me?**

**1) Yes (54.6%)	2) No	3) Don't know**

**4.5. Do your teachers mention their COI during their lessons?**

**1) Don't know	2) No (46.4%)	3) Some	4) All**

**Although it is valuable as an individual question, I realized that it is inappropriate to express it in the same sentence because 'professor' and 'courses' are not specified.**

**We revised the sentence as follows:**

**“A large number of students reported insufficient education on COI (63.7%), rarely attended lectures (8.5%), or conducted personal research (10.3%). Additionally, more than half (54.6%) of the respondents expressed interest in COI information about their courses.” [Revised Manuscript Line 131-134]**

-------------------------------------------------------------------------------------------------------------

(P6, lines 96-100 / para 4) This paragraph lists ratios of items, I don’t understand what this means, what is the ratio the authors are referring to? Do they just mean % of students who answered ‘yes’ to each question?

**To provide a clear depiction of the response rate to the questionnaire, we have revised the sentence and included a table for better understanding.**

**“****Upon comparing the results of previous studies conducted in France and Korea, the proportions of the three items****(3.3./3.4./4.6.) were similar, as depicted in the table 1 below. However, it was notable that French students expressed a significantly higher level of dissatisfaction regarding the adequacy of COI information(3.2./4.5).” [Revised Manuscript Line 139-143]**

**Table 1. Medical students' perceptions and experiences regarding COI information**

| **(% of students)** | **F** | **K** | **gab** |
| --- | --- | --- | --- |
| **3.2. During your studies, do you feel that you received enough**  **information about declaration of COI? (responding “No”)** | **85.2** | **63.7** | **-21.5** |
| **3.3. During your studies, did you receive a lecture or a tutorial on COI? (responding “Yes”)** | **4.3** | **8.5** | **4.2** |
| **3.4. During your studies, did you do any personal research (internet**  **for example) on the impact of COI or on the declaration of**  **COI? (responding “Yes”)** | **11.1** | **10.3** | **-0.8** |
| **4.6. I would like to know the COI of my teachers when they teach**  **me? (responding “Yes”)** | **66.6** | **54.6** | **-12.0** |
| **4.5. Do your teachers mention their COI during their lessons?**  **(responding “No”)** | **66.5** | **46.4** | **-20.1** |

-------------------------------------------------------------------------------------------------------------

(P8, line 114 / para 1) The authors report that ‘35.5% in Set 1 and 45.6% in Set 2 thought monetary offerings generated a COI’. A similar statement is repeated on p9,line 120 / para 1 and on p 13, line 152/para 1. I don’t understand this statement. I thought Set 1 and Set 2 were just groupings of questions, not groupings of participants?

**As the reviewer mentioned, Set1 and Set2 represent groups of questions. However, there was an error in expressing the analysis result.**

**We revised the sentence as follows:**

**“Give this, 35.5% in Set 1 and 45.6% in Set 2 considered monetary offerings as COI, demonstrating a higher awareness of direct offerings than indirect offerings.” [Revised Manuscript Line 159-161]**

**“Compared to the results of previous studies in France, Set 1 (34.5%) was at a similar level to Korea, but Set 2 (71.7%) showed a significantly higher level of awareness than Korean students.” [Revised Manuscript Line 166]**

-------------------------------------------------------------------------------------------------------------

(P11, line 142 / para 2) I suggest EDITING to read: “determine whether personal research on COI or lecture participation in COI affected BELIEFS ABOUT IMPACT OF COI ON future prescriptions BY SELF OR OTHERS more.”

(Table 3) I found this table very hard to interpret, and the summary sentence after it (beginning “the analysis found that the P-value…”) was also hard to understand. Can the authors please clarify the meaning of this table, perhaps providing the results in a different format would help?

**First, reflecting your opinion, we have made some changes to the format of Table 3 as shown below.**

**Beta is the data used to calculate the OR(Odds Ratio) and CI(Confidence Interval), and SD (Standard Deviation) represents the variability of the response. Therefore, these two factors are omitted as they do not hold significant meaning in interpreting the analysis results.**

**Furthermore, data points with p-values above the significance level (<0.05) are excluded, as they lack statistical significance.**

**Second, I have added the following explanation**

**Table 4. Medical students' perceptions of bias by class level.**

| **Question** | **Class level**  **(preclinical→clinical→resident)** | |
| --- | --- | --- |
|  | **p-value** | **OR 95% CI** |
| **Do you think COI can induce bias in drugs prescription for others?** | **0.007** | **1.76 (1.17~2.65)** |
| **COI: Conflict of interest**  **The reference category was the group of students answering “no” to the questions.**  **Note: The analyses were performed using gender as a covariate.** | | |

**“Table 3 demonstrates a 95% confidence interval indicating that for each increment in the class level, the likelihood of answering 'Yes' to that question increases by a minimum of 1.17 times and a maximum of 2.65 times.” [Revised Manuscript Line 194-197]**

-------------------------------------------------------------------------------------------------------------

(Table 4) I suggest the authors specify “Korean medical students” in the title, and can then delete the row of K K K K. This could be repeated in Table 5. I don’t understand the point of providing a P-value in this table or Table 5. I can’t see that it is relevant whether or not there is statistical significance between the ‘yes/no/don’t know’ groups.

(P 13, line 158) “The degree to which it affects…” Can the authors explain what they are talking about here?

(P14, line 167 / para 3) I suggest the following EDITS: “Regarding the STUDENTS’ PERCEPTION ABOUT possibility of bias…”

**We believe that the knowledge about COI is related to the importance of education. Therefore, even though it was not included in the previous study, we have included it as an additional aspect.**

**However, not only does the format match that of Tables 1 and 2, but there is no issue with conveying the meaning through a description instead of using tables. Therefore, Tables 4 and 5 will be omitted, and their contents will be discussed in the following section.**

**“Further analysis revealed that respondents who believed they had knowledge about COI (50.1%) exhibited a greater awareness of COI compared to those who believed they lacked knowledge (31.8%). Additionally, they demonstrated a higher awareness of direct COI (56.0%) compared to indirect COI (44.1%), as indicated in Table 1.” [Revised Manuscript Line 216-219]**

-------------------------------------------------------------------------------------------------------------

DISCUSSION

(P 15, line 179 / para 4) I suggest omitting this presentation of the paper as an hypothesis approach, as mentioned earlier.

**We have decided to understand and accept your opinion, so we omitted the sentence. [Revised Manuscript Line 235-236]**

-------------------------------------------------------------------------------------------------------------

(P 15, line 182 / para 5) Can the authors clarify their meaning: “We met the minimum study population of 95% confidence level …”

**As the answer to the question (P5, line 80-81 / para 4) above, the minimum number of participants was calculated with a confidence level of 95% and a margin of error of 5%, and the result was 380.**

**The number of participants in this study is 388, which meets this condition.**

-------------------------------------------------------------------------------------------------------------

(P 16, line 185-188) As mentioned above, I don’t think the authors can claim that this survey represents the opinions of Korean medical students.

**As the answer to the question (P5, line 80-81 / para 4) above, the minimum number of participants was calculated with a confidence level of 95% and a margin of error of 5%, and the result was 380.**

**The number of participants in this study is 388, which meets this condition.**

**As described in (P5, line 181-185) above, there is a limitation regarding participant concentration in specific areas and stages. However, despite these limitations, we believe the study still provides representation.**

-------------------------------------------------------------------------------------------------------------

(P 16, line 200 / para 5) The authors suggest students should spend more time on quality education about COI, but I wonder whether this is ignoring the responsibility of the educational institutions to encourage accurate and interesting education for students. Can the authors provide any evidence / references about this.

**We have included additional information regarding the challenges of medical ethics education for medical students in Korea, along with a relevant paper as a reference.**

**“****Currently, medical ethics education for medical students in Korea faces challenges due to an absolute lack of class time, insufficient presence of related scholars, unclear educational goals, and inadequate development of methodologies. Additionally, there is a lack of education specifically addressing COI (7) (8)”** **[Revised Manuscript Line 263-266]**

-------------------------------------------------------------------------------------------------------------

(P 17, line 17 / para 1) I really would like to see some stronger referencing of the literature on COI and suggests about systemic interventions that might reduce the influence of pharma reps / industry on students. This should go beyond individual disclosure / mandated transparency and might include topics such as : hospital / university policies to reduce pharma rep access to students, hospital / industry policies to limit the type of pharma rep gift giving and the amounts involved, university and hospital policies to limit the use of teachers who accept pharma industry gifts etc etc. Again, I encourage the authors to read widely around the topic of COI and how to reduce it so that they can provide a stronger argument and more useful suggestions for how Korea might address this issue in its healthcare system.

**Thank you for your suggestion and encouragement.**

**We deeply sympathize with the need for policies to reduce the impact of the pharmaceutical industry on medical students. Understanding the nature of COI and preparing policies to reduce biases caused by COI will be reflected in further follow-up studies.
Thank you again for your suggestion and encouragement.**

-------------------------------------------------------------------------------------------------------------

1.	JO S-G. A Study on the Rebate in the Medical Industry. Seoul Korea: Hanyang University Law School; 2020.

2.	Wazana A. Physicians and the Pharmaceutical IndustryIs a Gift Ever Just a Gift? JAMA. 2000;283(3):373-80.

3.	Ashley Wazana M. Physicians and the Pharmaceutical Industry Is a Gift Ever Just a Gift? JAMA. 2000;283(3):373-80.

4.	CHEONG Yoo-Seok PJ-H, Younsuck Koh. Ethical Issues Concerning the Relationship between Medical Students/Residents and the Pharmaceutical Industry. Korean J Med Ethics. 2011;14(2):215-23.

5.	Thompson DF. Understanding financial conflicts of interest. N Engl J Med. 1993;329(8):573-6.

6.	Medicine Io. Conflict of interest in medical research, education, and practice. Washington, D.C.: National Academies Press; 2009.

7.	Choi K. Medical Ethics and Professional Education: Educational Status and Roles of Philosophy. Journal of human studies. 2007;-(12):218-43.

8.	KWON I. A Critical Review of the Current Medical Ethics Education in Korea. Korean Journal of Medical Ethics. 2006;9(1):60-72.
