# Supplementary material for: Attitudes of medical students on conflict of interest: A comparative study of Korea and France
Source: PLoS One. 2023 Nov 2;18(11):e0293808. doi: 10.1371/journal.pone.0293808 (PMC10621834; doi:10.1371/journal.pone.0293808)
Supplement: Attachment — Submitted filename: 4. Response to Reviewers (Revision 2).docx [file pone.0293808.s004.docx]

**Response to Reviewers**

**Reviewers' comments:**

-------------------------------------------------------------------------------------------------------------------------

Abstract: This needs more work to act as a succinct representation of the paper. I suggest the first sentence should focus more on the problem – eg use line 43 in the paper: ‘Medical students are appealing marketing targets for pharmaceutical companies’ and then continue from line 19 about Etain’s study, and your replication of it. The background on Korean law (lines 16-19) is better kept in the paper alone. I would like a clear, succinct description of the method eg [1] an empirical survey study with Korean medical students concerning their educational experiences and views on conflicts of interest and [2] comparing and contrasting the results with Etain’s study of French medical students. The current method description (line 22-23) is very confusing – it suggests the authors are carrying out a review of ‘previous studies in Korea’ which is not correct. I found the wording of results in the Abstract (lines 24-25) was hard to follow. The authors might consider a revision such as: ‘Receipt of direct or indirect financial offerings from pharmaceutical industries was not properly recognised as COI by students.’ Finally, the last sentence in the Abstract should stick to the third person grammar and avoid using a second person approach like ‘your own’ (line 35).

**We revised the sentence as follows:**

**“****Medical students are potential marketing targets for pharmaceutical companies because established prescribing habits are not easily altered. In 2014, Bruno Etain and several other researchers published a paper which investigated the knowledge of and opinions on potential conflict of interest (COI) with regard to preclinical and clinical students enrolled in medical schools in France and residents working in hospitals. An empirical survey study with Korean medical students concerning their educational experiences and views on conflicts of interest and comparing and contrasting the results with Etain’s study of French medical students. Receipt of direct or indirect financial offerings from pharmaceutical industries was not properly recognised as COI by the medical students. Therefore, strengthening education on COI and implementing institutional improvements for COI disclosure are essential to prevent bias caused by COI and enhance awareness levels regarding COI.” [3. Manuscript (Revision 2) Line 16-26]**

-------------------------------------------------------------------------------------------------------------------------

Introduction

Line 40 I suggest pharma companies are not ‘compelled to’ engage in marketing, this word could be omitted

**We revised the sentence as follows:**

**“****For these reasons, pharmaceutical companies are conducting various direct and indirect marketing activities to influence doctors' prescriptions.” [3. Manuscript (Revision 2) Line 29-30]**

-------------------------------------------------------------------------------------------------------------------------

Line 43 The authors should repeat here the phrasing in the Abstract line 23 about ‘students, interns and residents, hereafter referred to as students’ – writing it in the Abstract alone is not sufficient

**We revised the sentence as follows:**

**“****Medical students, interns and residents (hereafter referred to as “students”) are potential marketing targets for pharmaceutical companies because established prescribing habits are not easily altered.” [3. Manuscript (Revision 2) Line 31]**

-------------------------------------------------------------------------------------------------------------------------

Line 52 I strongly disagree that disclosure is a solution for preventing conflicts of interest, but I recognise it is often reported this way in the literature. The authors could consider a comment about the controversy around this – eg others (myself included) regard disclosure as a necessary first step, although not sufficient for preventing COI. I expanded on this and other possible solutions in my previous review and the authors might find this reference helpful

Parker L, Karanges EA, Bero L. Changes in the type and amount of spending disclosed by Australian pharmaceutical companies: an observational study. BMJ Open 2019;9:e024928. doi:10.1136/ bmjopen-2018-024928

**Thank you for recommending the paper, and We have reviewed its contents in full.**

**As mentioned in the paper you recommended, regarding the importance of 'Transparency', 1) Transparency may assist those reading or receiving the disclosure to judge the risk of bias in those making the disclosure. 2) The act of disclosure may bring about changes where individuals refrain from engaging in related behaviors to avoid suspicions of bias arising. Therefore, in situations where voluntary disclosure is unlikely, We agree that measures are needed to compel the disclosure of conflicts of interest through the establishment of relevant laws and regulations, in order to prevent bias from occurring.**

**However, we aimed to approach conflicts of interest from an professional ethics perspective. Conflicts of interest are considered to be an moral issue for each individual, an professional ethics issue, and an issue related to human nature. Therefore, it can be relative depending on the differences in the individual's circumstances (e.g., income level, education level etc.) and their values (e.g., prioritizing professional integrity over financial benefits etc.).**

**Ethical issues cannot be perfectly controlled or resolved by regulatory means, as we believe that laws and policies are designed to restrict significant wrongdoings that exceed social standards and justice. The disclosure mentioned earlier in the document from the Institute of Medicine (IOM) is described as a "common solution" from this ethical perspective and appears to encompass a broader concept than disclosure from a legal and institutional standpoint. Therefore, in order to prevent bias in individuals facing conflicts of interest, there is a need for an ethical awareness of potentially risky situations, and various forms of education are essential to foster this correct awareness.**

-------------------------------------------------------------------------------------------------------------------------

Line 64 What is the difference between drug adoption case fees and drug prescription case fees?

**"drug adoption case fees" refers to case fees agreed upon for supplying pharmaceuticals to healthcare institutions and is also known as "landing fees."**

**"drug prescription case fees" refer to payments made in proportion to the prescription amount to induce the prescription of specific pharmaceuticals, and it is also referred to as "matching fees."**

-------------------------------------------------------------------------------------------------------------------------

Line 67 The paragraph here would be better moved to AFTER the paragraph at line 74. This would enable better flow of subjects in the text.

**We moved the paragraph [3. Manuscript (Revision 2) Line 58-64]**

-------------------------------------------------------------------------------------------------------------------------

Line 96 This was confusing. It needs to be re-worded as aims, not as hypotheses. I suggest editing to something like this (my suggested edits in capitals) : "[1] Korean students’ ABILITY TO identify some common situations as a potential COI; [2] Korean students REPORTS AND VIEWS ABOUT their education on COI; [3] Korean students PERCEPTIONS OF the bias induced by COI ON THEMSELVES AND OTHERS"

**We revised the sentence as follows:**

**“****This paper aims to identify the following: ① Korean students’ ability to identify some common situations as a potential COI; ② Korean students reports and views about their education on COI; ③ Korean students perceptions of the bias induced by COI on themselves and others.” [3. Manuscript (Revision 2) Line 78-80]**

-------------------------------------------------------------------------------------------------------------------------

Results

Line 141 The authors report French and Korean student answers to item 4.6 are similar, but Table 1 shows they are quite different (66.6 % vs 54.6%)

**We revised the sentence as follows:**

**“Upon comparing the results of previous studies conducted in France and Korea, the proportions of the two items(3.3./3.4.) were similar, as depicted in the table 1 below.” [3. Manuscript (Revision 2) Line 112]**

--------------------------------------------------------------------------------------------------------------

Line 143 student dissatisfaction with COI information is more properly shown in item 4.6 than 4.5

Table 1, I don’t understand what ‘gab’ refers to (head in final column)

**We revised the sentence as follows:**

**“However, it was notable that French students expressed a significantly higher level of dissatisfaction regarding the adequacy of COI information(3.2./****4.6.)” [3. Manuscript (Revision 2) Line 114]**

**We also used the term "gab" to refer to the difference between numbers compared in a statistical table, but We realized that it was not an appropriate expression. The term "gab" has been changed to "difference." [3. Manuscript (Revision 2) Line 115 Table 1]**

-------------------------------------------------------------------------------------------------------------------------

Line 146-147 I don’t understand what this sentence is about, can the authors reword to clarify.

**Lines 146-147 aimed to convey the response to the question, "Do you think you know how to define what a COI is?", and the responses were as follows.**

**We revised the sentence as follows:**

**“****In response to the question, "Do you think you know how to define what a COI is?", only 24.8% responded that they could define it, and there was no significant difference by class level (PC: 26.8%; C: 25.9%; R: 32.1%; P = 0.1385). Compared to the results of a previous study in which 64.6% of French students claimed they could define it, Korean students' knowledge about COI was found to be low (24.8%).” [3. Manuscript (Revision 2) Line 117-121]**

-------------------------------------------------------------------------------------------------------------

Line 150 Do the authors mean to say that 51 students answered yes to BOTH questions, or to at least one of the questions listed? And when they say 23 out of 51 respondents declared they cannot define COI, is this the same 51 who answered yes to both (or at least one of?) the questions listed?

**We revised the sentence as follows:**

**Students who answered "Yes" to at least one of the following two questions were 51 (13.1%): "Do you think you received enough information about COI disclosure?" and "Have you ever received a special lecture or individual training on COI?”. Furthermore, out of the 51 respondents who answered "Yes" to at least one of the questions, 23 (45.1%) of them responded with "No" or "I'm not sure" to the question "Do you think you know how to define what a COI is?", indicating that COI education is not being properly conducted. [3. Manuscript (Revision 2) Line 122-127]**

-------------------------------------------------------------------------------------------------------------------------

Table 2 and 3 I still don’t understand the p value. Is it reporting homogeneity between preclinical, clinical and residents within one country, or is it reporting homogeneity between preclinical in France and Korea; clinical in France and Korea, and residents in France and Korea? Neither seem to be feasible, given the variation in results so more information is needed about the figures in order to convince me that they are correct.

**We should consider the p-values for France and Korea independently in Table 2 and Table 3. In other words, it represents the homogeneity of responses based on class level in each case for France and Korea, respectively.**

**Additionally, it is expected that the reviewer interprets lower p-values in Table 2 as indicating higher homogeneity. (Hence the numerical discrepancy you mentioned.)**

**However, our conducted chi-square test has the following null and alternative hypotheses, and if the p-value is less than the significance level (0.05), we reject the null hypothesis (H0) and accept the alternative hypothesis (H1).**

**Null Hypothesis (H0): There is no difference in responses based on class level. Alternative Hypothesis (H1): There is a difference in responses based on class level.**

**In other words, the lower the p-value, the lower the homogeneity of responses, and the higher the p-value, the higher the homogeneity.**

**Therefore, if the p-value is below the significance level, we can interpret it as a statistically significant difference in responses based on class level.**

**Furthermore, in the case of Table 3, it is used to determine whether the logistic regression results are statistically meaningful, regardless of homogeneity.**

-------------------------------------------------------------------------------------------------------------------------

Line 177 It seems that Table 4 and associated text should be put here

**We moved the paragraph [3. Manuscript (Revision 2) Line 162-165]**

-------------------------------------------------------------------------------------------------------------------------

Line 183 I don’t understand this comment, please edit to clarify

**We revised the sentence as follows:**

**“****In particular Table 3, the responses to the question "Have you ever received a gift from the PI?" showed the largest difference between Korean (15.7%) and French (62.7%) students, while the responses to the question "I consider it as a COI when attending a meal sponsored by the PI" showed the smallest difference between Korean (17.3%) and French (21.4%) students.” [3. Manuscript (Revision 2) Line 170-173]**

-------------------------------------------------------------------------------------------------------------------------

Line 218 Who are ‘they’?

**We moved the paragraph and revised the sentence as follows:**

**“****In the additional analysis of Table 2, we applied the responses to the question "Do you think you can define what a conflict of interest is?" instead of the responses to the question "What is your class level?" The results showed that respondents who answered "Yes" had a higher awareness of COI (50.1%) compared to those who answered "No" (31.8%). Furthermore, among the respondents who answered "Yes," the awareness of direct COI (Set 2) (56.0%) was higher than the awareness of indirect COI (Set 1) (44.1%). “ [3. Manuscript (Revision 2) Line 145-150].**

-------------------------------------------------------------------------------------------------------------------------

Line 220 I don’t understand this comment, please edit to clarify

**Further analysis of Table 3, we also examined the responses to the question "Do you think you can define what a conflict of interest is?" instead of the question "What is your class level?" As a result, the overall percentage of respondents who answered "Yes" (37.9%) and "No" (40.1%) was similar. Furthermore, among those who answered "Yes," the percentage of those who believe that others can develop bias (68.2%) was higher than the percentage of those who believe they can develop bias (14.2%). [3. Manuscript (Revision 2) Line 174-179].**

-------------------------------------------------------------------------------------------------------------------------

Discussion

Line 232 can the authors provide a 1-2 line summary of the comparative results here?

**We believe the summary of the requested comparative results is described in the paragraph just before Line 228. Therefore, We have moved Line 232 paragraph forward and made some modifications to its content. :**

**“****Additionally, while each study conducted in France and Korea was not specifically designed to demonstrate differences, it would be meaningful to compare the research findings between the two countries using a consistent approach to validate any potential disparities.” [3. Manuscript (Revision 2) Line 182-185].**

-------------------------------------------------------------------------------------------------------------------------

Line 237 onwards. The limitations section needs work. The comment about getting to statistical significance is not relevant in relation to % of the student population surveyed. The study has plenty of merit so I suggest editing this section, starting with the study strengths (diversity of participants at all levels – students, interns, residents; even participation from a broad range of training departments ie 18/26) and then acknowledge study limitations (the majority being clinical; most concentrated in just a few regions). As previously mentioned in my original review, the authors cannot assume that the study represents the opinions of the majority of Korean medical students because they do not know the number of total students in the country. I would omit that paragraph starting 244.

**We revised the sentence as follows:**

**However, The main potential limitation of this study is the lack of representation, which is also showed in previous studies. The majority of respondents were locally concentrated in some regions (Seoul, Gyeonggi, Gangwon), with more than half of them being enrolled at the clinical level (PC: 14.4%; C: 64.6%; R: 20.8%). This imbalance is expected to reflect the following reasons: The survey on COI for students in Korea is the first attempt. In addition, many surveyed organizations were reluctant to distribute the questionnaire due to lack of understanding of COI and administrative burden.” [3. Manuscript (Revision 2) Line 199-204].**

-------------------------------------------------------------------------------------------------------------------------

Line 254 onwards This paragraph discussing the results would be better moved up earlier in the Discussion, after paragraph 3 of the Discussion (ie before the Limitations section)

**We moved and revised the paragraph as follows :**

**“****The strengths of this study include the diverse participation of medical students, interns, and residents at all stages of medical education. In particular, residents evenly participated in 18 out of the 26 training departments operating in Korea, with the exception of 5 departments with a total quota of less than 50.” [3. Manuscript (Revision 2) Line 195-198].**

-------------------------------------------------------------------------------------------------------------------------

Line 261 I don’t understand the comment/interpretation that this study shows students’ have a ‘basic sense of ethics.’ Can the authors edit to clarify or omit.

**It is common to believe that under the same conditions of a COI situation, the likelihood of bias occurring is the same for oneself as it is for others. However, thinking that even though bias is likely to occur in others, the likelihood of bias occurring in oneself is low can be seen as an expression of one's determination not to go in the wrong direction. This is what we have referred to as a "basic sense of ethics."**

-------------------------------------------------------------------------------------------------------------------------

Line 267 Can this authors justify this paragraph / comment, eg with reference to the literature.

**In Korea, not only is there a shortage of research literature on COI, but this is even more pronounced when it comes to education about COI. However, it is worth noting that, as an alternative to research literature, there are currently no courses dedicated to the topic of COI in the curriculum of Korean medical schools.**

-------------------------------------------------------------------------------------------------------------------------

Line 270 As mentioned, I disagree that COI disclosure will prevent bias, but I acknowledge that what the authors have written is a widely held view.

-------------------------------------------------------------------------------------------------------------------------
